# Effects of food resources on the fatty acid composition, growth and survival of freshwater mussels

**DOI:** 10.1371/journal.pone.0173419

**Published:** 2017-03-07

**Authors:** Michelle R. Bartsch, Lynn A. Bartsch, William B. Richardson, Jon M. Vallazza, Brenda Moraska Lafrancois

**Affiliations:** 1 United States Geological Survey, Upper Midwest Environmental Sciences Center, La Crosse, Wisconsin, United States of America; 2 National Park Service, Ashland, Wisconsin, United States of America; University of Wisconsin Milwaukee, UNITED STATES

## Abstract

Increased nutrient and sediment loading in rivers have caused observable changes in algal community composition, and thereby, altered the quality and quantity of food resources available to native freshwater mussels. Our objective was to characterize the relationship between nutrient conditions and mussel food quality and examine the effects on fatty acid composition, growth and survival of juvenile mussels. Juvenile *Lampsilis cardium* and *L*. *siliquoidea* were deployed in cages for 28 d at four riverine and four lacustrine sites in the lower St. Croix River, Minnesota/Wisconsin, USA. Mussel foot tissue and food resources (four seston fractions and surficial sediment) were analyzed for quantitative fatty acid (FA) composition. Green algae were abundant in riverine sites, whereas cyanobacteria were most abundant in the lacustrine sites. Mussel survival was high (95%) for both species. *Lampsilis cardium* exhibited lower growth relative to *L*. *siliquoidea* (p <0.0001), but growth of *L*. *cardium* was not significantly different across sites (p = 0.13). In contrast, growth of *L*. *siliquoidea* was significantly greater at the most upstream riverine site compared to the lower three lacustrine sites (p = 0.002). *In situ* growth of *Lampsilis siliquoidea* was positively related to volatile solids (10 – 32 μm fraction), total phosphorus (<10 and 10 – 32 μm fractions), and select FA in the seston (docosapentaeonic acid, DPA, 22:5n3; 4,7,10,13,16-docosapentaenoic, 22:5n6; arachidonic acid, ARA, 20:4n6; and 24:0 in the <10 and 10 – 32 μm fractions). Our laboratory feeding experiment also indicated high accumulation ratios for 22:5n3, 22:5n6, and 20:4n6 in mussel tissue relative to supplied algal diet. In contrast, growth of *L*. *siliquiodea* was negatively related to nearly all FAs in the largest size fraction (i.e., >63 μm) of seston, including the bacterial FAs, and several of the FAs associated with sediments. Reduced mussel growth was observed in *L*. *siliquoidea* when the abundance of cyanobacteria exceeded 9% of the total phytoplankton biovolume. Areas dominated by cyanobacteria may not provide sufficient food quality to promote or sustain mussel growth.

## Introduction

The dietary requirements of freshwater unionid mussels that are essential for their growth, reproduction and survival are poorly understood. In laboratory growth studies, Gatenby et al. [1] suggested that juveniles require a diet containing mixtures of algae, particularly those high in polyunsaturated fatty acids. They also suggested that the presence of fine sediment (<130 μm) facilitates pedal feeding and the collection of food particles (i.e., live algae) and that bacteria were not essential to the growth and survival of newly metamorphosed juvenile mussels (*Villosa iris* and *Pyganodon grandis*; [2]). In contrast, others have found strong evidence to suggest bacteria are an important component in unionid diets, particularly for adults [3, 4]. Several laboratory studies have addressed particle-retention efficiencies of both freshwater and marine mussels. Vanderploeg et al. [5] documented that bivalves (*Lampsilis radiata siliquoidea*) had significant retention efficiencies for very small particles in seston <1 μm, suggesting they can filter nearly all sizes of algae, including algal picoplankton. However, the relationship between food consumption and assimilation remains under investigation. Nichols and Garling [3] showed through stable isotope analysis that the main dietary item for seven species of adult unionids in a detrital-rich river and an algal-rich lake was bacterial and not an algal-derived food source. Christian et al. [4] also found that a large portion of the diet of two mussel species, *Elliptio dilatata* and *Ptychobranchus fasciolaris*, originated from the living microbial biomass associated with the fine particulate organic matter (operationally defined as particles ranging in size from 0.9 to 250 μm). Although ratios of stable isotopes of carbon and nitrogen have been used to determine food sources of unionids, the interpretation of these ratios is often difficult and ambiguous. Stable isotopes track only stored compounds [3] and rely on distinct differences in isotopic ratios between food items to help determine food use [6].

Quantitative fatty acid (FA) analysis has emerged as a promising method to distinguish both food quality and quantity in aquatic food webs [7]. The essential fatty acids (EFAs) are those defined as necessary for cellular function and organismal health, and are only produced by autotrophs and bacteria [8]. Lack of adequate intake of EFAs can result in poor growth and survival and is reflected in population declines in a variety of organisms [8]. Marine mussels have shown limited capacity to synthesize *de novo* polyunsaturated fatty acids (PUFAs) [9] and their FA composition is shown to closely match that of their diets [10, 11]; however, little is known about the *in situ* diets and associated FA composition of freshwater bivalves. Budge et al. [11] documented high PUFA levels (~50% of total fatty acids) in *Mytilus edulis*, with 20:5n3 (eicosapentaenoic acid, EPA) and 22:6n3 (docosahexaenoic acid, DHA) comprising approximately 30% of the total FA composition. They also found that proportions of 20:4n6 (arachidonic acid, ARA) were five-fold greater in mussels compared to phytoplankton from the same location, suggesting that the mussels were selectively retaining this FA [11]. Identifying the composition of various food resources and the extent of selective feeding by freshwater mussels could provide insight into their essential dietary requirements.

The St. Croix River (MN, WI) is often recognized for its good water quality and features a robust native mussel fauna including 40 mussel species, of which nearly half are state or federally endangered or threatened. The river has been the subject of intensive research, monitoring, and management due to concerns about increased urbanization and agricultural activity. A paleolimnological study found that phosphorus loading rates to Lake St. Croix, a natural riverine impoundment at the lower end of the river, were four times higher than before European settlement [12]. Although phosphorus and sediment inputs to Lake St. Croix decreased from 1976 – 2004, nitrate inputs have increased [13] and dramatic changes in algal communities have occurred, with a marked shift from benthic to more planktonic species [14]. Bloom-forming cyanobacteria are now common in late summer throughout Lake St. Croix [15]. In addition, there have been documented declines in juvenile mussel recruitment below St. Croix Falls and it is speculated that these declines may be related to changes in sediment composition [16]. Raikow and Hamilton [17] hypothesized that changes in sediment type affects the composition of the microbial assemblages in sediments, which in turn affects benthic food quality or palatability. An incomplete understanding of the mechanisms causing the decline in juvenile recruitment underscores the overall lack of knowledge of food requirements of unionid mussels.

Despite the breadth of unionid mussel research, there is little understanding of how changes in water quality affect food resources critical for the survival and growth of native mussels. Given the nutrient enriched conditions in the St. Croix River, particularly within Lake St. Croix [14], it is likely that the system has moved toward a state of lowered food quality. Recent research suggests that Lake St. Croix is dominated by cyanobacteria, especially during low flow years, and that their abundance and composition vary spatially from upstream to downstream [15]. Increased densities of cyanobacteria and change in species distribution may have important implications for variation in planktonic food quality, such that as cyanobacteria become more abundant in the phytoplankton community, juvenile mussel growth may be reduced, as was indicated for other freshwater bivalves [18]. This study (1) characterized the relationships between nutrient conditions and mussel food quality (seston and benthic sediments) in the St. Croix River and (2) experimentally investigated the *in situ* effects of food quality (seston and benthic sediments) on the fatty acid composition, growth and survival of juvenile mussels in the river corridor and the four sub-basins of Lake St. Croix.

## Material and methods

### Ethics statement

A scientific research and collection permit was obtained through the National Park Service in order to conduct this study within the St. Croix National Scenic Riverway Park. No additional permits were required for the sample collection described herein. No threatened or endangered species were collected as a part of this study.

### Study area

**Fig 1:**
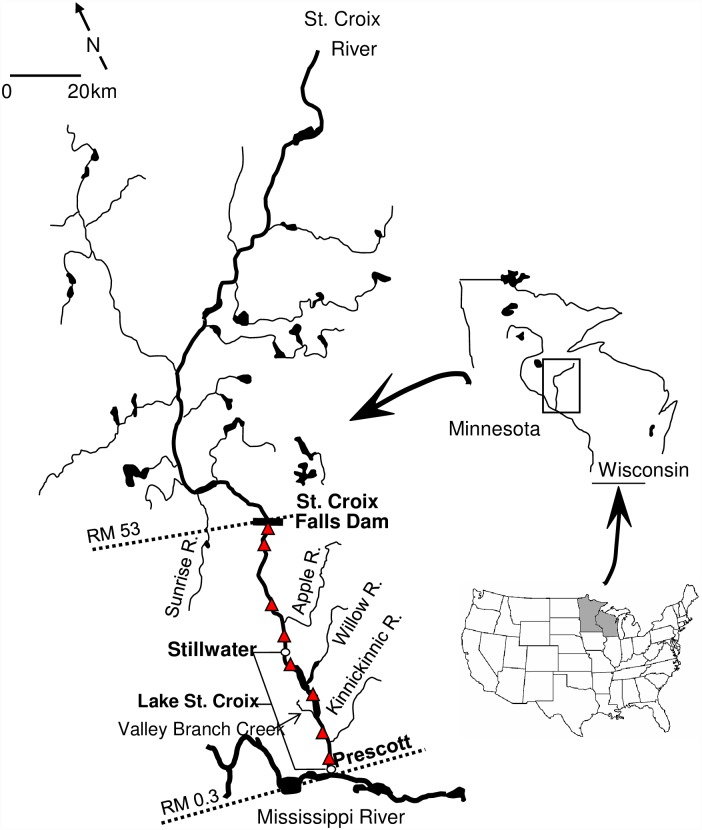
Sampling sites (red triangles) within the St. Croix National Scenic Riverway.

The St. Croix River forms the northern border between Wisconsin and Minnesota, USA, and terminates at the confluence of the Mississippi River, near Prescott, WI (Fig 1). Our study area encompassed the lower 53 km of the river from St. Croix Falls Dam to the confluence of the Mississippi. The lowermost 40 km of the St. Croix River function as a lake, Lake St. Croix, due to its impoundment by the Mississippi River. Lake St. Croix is divided into four sub-basins by the deltas of the entering tributaries: the Willow River (RM18), Valley Branch Creek (RM11) and the Kinnickinnic River (RM6, Fig 1). Our eight study sites included four riverine sites located below St. Croix Falls and four lacustrine sites located in Lake St. Croix, with one site in each of four sub-basins; all sites were known to contain mussels. These sites have been characterized by previous research [12, 13] to represent contrasting water quality conditions and phytoplankton communities that may potentially affect available food quality (seston and benthic) and ultimately affect juvenile mussel survival and growth.

### Test organisms

*Lampsilis cardium* and *L*. *siliquoidea* were selected for this study because they have a broad geographical distribution and they are congeners of the federally endangered *L*. *higginsii*, which also occurs within this system. Sexually immature juveniles were chosen because of their limited food reserves and the likelihood that they would expend their energy for growth and not reproduction. The juvenile mussels (1 to 2 yr old) were provided by the Genoa National Fish Hatchery, Genoa, Wisconsin and transferred to the U.S. Geological Survey, Upper Midwest Environmental Sciences Center (UMESC) in La Crosse, Wisconsin. The juveniles were held in a flow-through raceway containing well water (15 ± 1.7°C) and fed a commercial diet of *Nannochloropsis* (Nanno 3600, strain: CCMP525; 8 mL/d; 1-2 μm cell size; Reed Mariculture, Campbell, CA, USA) for 38 d prior to deployment in the St. Croix River. During this holding period, subsamples of the juveniles (n = 5 of each species) were submitted to the U.S. Fish and Wildlife Service, La Crosse Fish Health Center (Onalaska, WI) for testing of common fish pathogens. Both species were negative for the tested pathogens, including viral hemorrhagic septicemia virus (VHS). Juvenile foot tissue from two individuals of each species was also collected to provide an initial fatty acid profile. Each tissue sample was transferred to a cryovial, then flash-frozen in liquid nitrogen, and stored in an ultracold freezer (-80°C) until being freeze-dried prior to fatty acid analyses.

In addition to the field deployed juveniles, subsamples of both species (n = 6 *L*. *cardium* and n = 5 *L*. *siliquoidea*) were concurrently maintained in the flow-through, laboratory raceway containing well water (held at 15 ± 0.5°C) and fed the commercial, *Nannochloropsis* diet until the termination of the *in situ* field test. At the end of 28 d field deployment, foot tissue from the laboratory-held juveniles was then collected to provide an estimate of diet source retention, by comparing the net change in the tissue fatty acid to that available in the *Nannochloropsis* diet. We expressed this tissue to diet change as fatty acid accumulation ratios (FAAR) for each of the polyunsaturated fatty acids similar to Bӧhm et al. [19] as follows.

$$FAAR=\frac{Net\;tissue\;change\;in\;ith\;fatty\;acid}{ith\;fatty\;acids\;in\;the\;food}$$

Comparisons were based on mass fraction ratios in the mussel foot tissue to that of the commercial diet.

### *In situ* juvenile mussel exposures

A 28 d *in situ* juvenile mussel exposure was conducted from July 22 to August 19, 2008 at the eight study sites during a typical, low flow summer (~115 m^3^ s^-1^, based on an 8 y median flow; USGS 05344490 St. Croix River at Prescott, WI). Juveniles were randomly deployed into six *in situ* exposure cages constituting two treatments, (1) three cages suspended midway in the water-column (~1-m depth) and (2) three placed at the sediment-water interface, with two juveniles per cage, one of each species. Juveniles were suspended midway in the water-column to prevent contact with sediment because mussels have been shown to obtain food resources from sediments through pedal feeding [17, 20]; and therefore, we could compare their growth response to resources either associated primarily with the water-column or the sediment. The cages were constructed of polyethylene mesh cylinders (1.3 cm mesh, 25.4 x 10.2 cm diameter), with 10.2 cm diameter polyvinyl chloride drain grates on the top and bottom, and were attached to 92-cm fence posts with stainless steel hose clamps. Prior to deployment, the initial lengths (nearest 0.01 mm) of each juvenile were recorded using a Digimatic caliper (Model 500–172, Mitutoyo/MTI Corporation, Tokyo, Japan). To reduce the variation in growth measurements, juvenile lengths were measured by one individual. Precision (relative standard deviation) of the length measurements, estimated from analysis of 10 juveniles (6 *L*. *cardium* and 4 *L*. *siliquoidea*) which were measured five times each, ranged from 0.13 to 0.78%. Upon test termination, each juvenile mussel was recorded as living (firmly closed shell) or dead (empty shell or continued shell gap upon probing) and the growth of live individuals was assessed by measuring shell length (mm). Each live juvenile was sacrificed by severing the adductor muscle and the foot tissue was removed, transferred to a cryovial, then flash-frozen in liquid nitrogen, and stored in an ultracold freezer (-80°C) until being freeze-dried prior to fatty acid analyses.

At the mid-point of the *in situ* deployment (12 – 15 d), water (seston) and surficial sediment samples were collected for food resource analysis. Near each cage deployment site, water samples were collected at 0.5 m depth using acid-washed 1-L polypropylene bottles and stored in coolers on ice prior to being processed. We filtered water from a given site through Nitex^™^ sieves to obtain three size fractions (<63 μm, <32 μm, <10 μm) and a whole water sample. Previous studies indicated that the <28 μm size fraction is typically ingested by adult unionid mussels [3]. Seston fatty acid samples were processed by filtering 1.0 – 3.4 L of screened, pooled water for each of the four fractions onto unashed, Pall GF/F filters (Type A/E, 0.9 μm, 47-mm diameter, product no. 61631, Pall Corporation, Port Washington, NY). Additionally, three surficial sediment samples (top 1 cm) were collected with a 5-cm diameter core at each site. Each filter and sediment sample was transferred to a cryovial, flash-frozen in liquid nitrogen, and then stored in an ultracold freezer (-80°C) prior to fatty acid extraction.

### *In situ* site characteristics

Water temperature was continuously monitored (at 15 min intervals) using Thermochron iButton temperature loggers (Maxim Integrated Products, Inc., Sunnyvale, CA, USA) at all eight locations and both positions (water column and sediment-water interface) throughout the experiment (Table 1). In addition, water quality monitors (YSI 600 XLM multi-parameter water quality probes, Yellow Springs, OH, USA) were deployed at six sites (two riverine, the upper and lower most extent, and all four sub-basins of Lake St. Croix) to continuously measure dissolved oxygen, pH, specific conductance, and temperature during the last two weeks of mussel cage deployment (Table 1).

**Table 1:** Water quality characteristics (mean ± 1 SE) at four riverine (R1-R4) and four lacustrine (L5-L8) sites on the St. Croix River during a 28 d *in situ* exposure.

	iButton temperature (°C)^a^		Water quality probe^b^			
Site	Upper	Lower	Temperature (°C)	Dissolved oxygen (mg/L)	Conductivity (μS/cm)	pH
R1	24.5 ± 0.02	23.6 ± 0.04	23.6 ± 0.04	8.1 ± 0.03	208 ± 0.2	8.02 ± 0.01
R2	24.6 ± 0.02	24.6 ± 0.02	— ^c^	—	—	—
R3	24.7 ± 0.02	24.9 ± 0.02	—	—	—	—
R4	25.0 ± 0.02	24.9 ± 0.03	24.1 ± 0.05	8.5 ± 0.04	224 ± 0.2	8.15 ± 0.01
L5	25.6 ± 0.02	25.8 ± 0.02	25.3± 0.04	9.5 ± 0.08	226 ± 0.2	8.25 ± 0.02
L6	25.8 ± 0.02	24.5 ± 0.02	25.2 ± 0.04	9.7 ± 0.06	238 ± 0.4	8.26 ± 0.01
L7	25.7 ± 0.01	25.8 ± 0.01	25.2± 0.02	10.0 ± 0.04	225 ± 0.0	8.40 ± 0.01
L8	25.3 ± 0.02	25.4 ± 0.02	24.8 ± 0.03	8.7 ± 0.05	231 ± 0.2	8.33 ± 0.01

^a^Thermochron iButton temperature loggers continuously monitored water temperature at 15 min intervals within the water column (upper) and at the sediment-water interface (lower) at each site for 28 d.

^b^YSI 600 multi-parameter water quality probes deployed the last 14 d of mussel cage deployment.

^c^Water quality probes not deployed.

Seston samples in each of the four fractions (200 – 1000 mL of whole water, <63μm, <32 μm, or <10 μm onto pre-combusted Pall GF/F filters) were used for the determination of total suspended and volatile solids [21]. Water for each of four fractions (200 mL) was filtered onto Pall GF/F filters and analyzed spectrophotometrically for chlorophyll *a* [21] using a Beckman DU 640 (Brea, CA, USA). Filtered seston samples from each of the four fractions (1.0 – 3.3 L) were also analyzed for carbon and nitrogen content on an ECS 4010 CHNSO elemental combustion analyzer (Costech Analytical Technologies Inc, Valencia, CA, USA). Nutrient samples (60 mL for each of four fractions, acidified to a pH ≤2 with concentrated sulfuric acid) were analyzed for total phosphorus (TP) and total nitrogen (TN) using persulfate digestion followed by the ascorbic acid method for TP and the hydrazine reduction method for TN [21] with a Konelab 30 discrete autoanalyzer (Konelab Corporation, Espoo, Finland). Soluble reactive phosphorus (SRP, 60 mL of whole water, filtered through Pall GF/F) was also analyzed using the ascorbic acid method [21]. Whole water samples (60 mL, filtered and acidified to a pH ≤2 with concentrated sulfuric acid) were analyzed for nitrate-nitrite nitrogen (NO_3_-NO_2_) using the cadmium reduction method and for ammonium nitrogen (NH4^+^-N) using the phenate method [21] with a Techicon^®^ continuous-flow autoanalyzer (Seal Analytical, Mequon, WI, USA). Dissolved inorganic nitrogen (DIN) was also reported, which is the sum of nitrate-nitrite and ammonia nitrogen. All samples were collected and analyzed in duplicate.

Members of the ambient algal community were identified (usually to species [22–32]) at each site using whole water samples (100 mL) that were collected and preserved in Lugol’s solution. Phytoplankton were identified and enumerated in 10 mL settling chambers using a Nikon Diaphot phase inverted microscope (Melville, NY), following the Utermöhl [33] sedimentation method. A minimum of three hundred biological units or eight grids were counted per sample [34]. A maximum of 100 grids were counted in instances where phytoplankton density was extremely low (<3 biological units per grid; [34]). Biovolume was calculated using geometric models described in Hillebrand et al. [35] and Sun and Liu [36].

Three sediment samples (top 1 cm) were collected from cores and analyzed for carbon and nitrogen on a vario Max high temperature combustion C/N analyzer (Elementar Inc, Hanau, Germany). Sediment pore water was pumped from sediments surrounding the cages using a mini drivepoint sampler with a single inlet, positioned at 2.5 cm and connected to a variable-speed peristaltic pump (Geotech Environmental Inc., Denver, CO, USA) equipped with a single pump head (Masterflex L/S 7518–10) pumped at rate ~10 mL/min [37]. The pore water samples (n = 3 per site) were analyzed for total ammonia nitrogen (TAN) with the automated phenate method [21] on a Technicon^®^ continuous-flow analyzer (Seal Analytical, Mequon, WI, USA). Concentrations of unionized ammonia (NH_3_-N) were calculated from site- and replicate-specific ambient TAN, pH, and temperature measurements with the formula of Emerson et al. [38].

### Fatty acid analyses

Total lipids were extracted from freeze-dried juvenile foot tissue according to methods described by Hebert et al. [39]. Fatty acids were extracted from freeze-dried juvenile foot tissue, seston, and surfical sediment samples and methylated according to methods described by Hebert et al. [39]. Fatty acid methyl esters (FAME) were analyzed using a gas chromatograph (Agilent 7890, Wilmington, DE, USA) equipped with flame ionization detector (FID) and a Supelco 2560 capillary column (100 m X 0.25 mm with 0.2-μm film thickness; Supleco, Bellefonte, PA, USA). Samples (0.5 μL) were injected into the gas chromatogram and helium was used as the carrier gas. The following temperature program was employed: 140°C held for 5 min, then heated 4°C per minute to 240°C, held for 15 min. The FID was held at 260°C while the injector was maintained at 250°C. Individual FAs were identified using Supleco’s 37 component FAME standard (#47885-U; Sigma-Aldrich, St. Louis, MO, USA) by comparing peak retention times between samples and standards dissolved in n-hexane. The method retention times were locked using 18:0 at 26.5 min. An internal standard (5α-cholestane; Sigma-Aldrich; #C8003) was added to the sample tissue before extraction to estimate percent recovery. The FAME standard was run as a 5-point standard curve and used to quantify the amount of each acid within a set of twenty samples. Additional single FA standards were used to expand the range of quantifiable FAME to include other important FA (i.e., vacenic acid, 18:1n7; docosapentaeonic acid, DPA, 22:5n3; 7,10,13,16-docosatetraenoic, 22:4n6; 4,7,10,13,16-docosapentaenoic, 22:5n6) not included in the FAME 37 standard. Fatty acid results were reported as μg FAME/mg dry weight for mussel foot tissue, as μg FAME/L for seston, and as μg FAME/g dry weight for sediment samples. We calculated the sum of all omega-3 to the sum of omega-6 (i.e. n3:n6) FA ratio, which has been suggested as an indicator of the amount of autochthonous (aquatic algae) versus allochthonous (terrestrial) food sources in the diet [40].

To assess the importance of bacterial FAs as a source of nutrition for juveniles, a sub-sample of mussel foot tissue (from each position), seston (from four fractions) and surficial sediment from each site were analyzed for bacterial fatty acid methyl esters (BAME). Sample BAMEs were analyzed using a gas chromatograph (Agilent 6890, Wilmington, DE, USA) equipped with flame ionization detector (FID) and a Supelco SPB-1 capillary column (30 m X 0.25 mm with 0.25-μm film thickness; Supleco, Bellefonte, PA, USA). Samples (0.5 μL) were injected into the gas chromatogram and helium was used as the carrier gas. The following temperature program was employed: 50°C held for 2 min, then heated 50°C per minute to 150°C, then heated 3°C per minute to 250°C, held for 21 min. The FID was held at 280°C while the injector was maintained at 250°C. Individual FAs were identified based on the retention times of BAME standards (BAME mix Supleco; #47080-U; Sigma-Aldrich) dissolved in n-hexane and quantified using a 5-point standard reference calibration curve. Additional bacterial FAs were used to expand the range of quantifiable BAME to include potentially important FAs (i.e., 12-methyltridecanoic acid [iso, 14:0iso]; 13-ethylpentadecanoic acid [anteiso, 16:0ai]; 14-methylhexadecanoic acid [anteiso, 17:0ai]) not included in the BAME mix. The method retention times were locked using 16:0 at 23.5 min. Bacterial FA results are reported as μg BAME/mg dry weight of mussel foot tissue, as μg BAME/L for seston, and as μg BAME/g dry weight for sediment samples. We defined bacterial derived FAs as the sum of 14:0iso, pentadecanoic acid (15:0), 13-methyltetradecanoic acid (iso, 15:0i), 12-methyltetradecanoic acid (anteiso, 15:0ai), 14-methylpentadecanoic acid (iso, 16:0i), 16:0ai, 15-methylhexadecanoic acid (iso, 17:0i), 17:0ai, and heptadecanoic acid (17:0) [11].

### Statistical analysis

Partial Least Squares Regression (PLSR, SAS/STAT^®^ 9.2 2008) was used to identify variables that best predict differences in the observed juvenile growth relative to sites. Potential predictor variables of growth included water column nutrients, seston variables measured on size fractions (chlorophyll *a*, total suspended solids, volatile suspended solids, and FAs), and sediment nutrients, carbon, and FA contents. Variables were selected based on Wold’s [41] variable importance for projection (VIP) criterion of >1.25 [42]). The PLSR is an extension of multiple linear regression where the associations of predictor variables to dependent variables are extracted through a series of latent factors that maximize the explained variance in the dependent variable(s). These linear combinations (factors) are based on centered and scaled (mean = 0, var = 1) predictors. The influence of each predictor variable is independent of one another because its effect is expressed as a factor loading rather than a coefficient as in the multiple regressions (which is dependent on the complement of variables specified in the model). In the PLSR, the first factor describes the largest proportion of explained variance, with each additional factor describing a successively smaller proportion. The reduction of multi-dimensionality through PLSR results in a lower number of orthogonal latent factors which are used to detect structure in the data. The PLSR approach is more appropriate than multiple regression (MR) when 1) collinearity is suspected among predictor variables (i.e., X variables are correlated among themselves), and 2) when the number of measured variables exceeds the number of observations. Multiple regression would limit the number of variables in the model to a maximum of seven in this case given that there are eight observations (sites). Further, PLSR has been shown to be more reliable in identifying relevant variables and their influence when small sample size and collinearity is suspected [43].

Linear discriminant analysis was used to identify a subset of FAs that could successfully differentiate individual sites for a given mussel species. This analysis was judged appropriate given the common covariance matrix (i.e., sites) for all groups (JMP version 10, SAS Institute Inc., Carry, NC, USA). Mahalanobis distance measure was used to calculate the individual observation distance from the group multivariate mean. Mahalanobis distance accounts for the variance of each variable and the covariance between variables and provides a relative measure of distance that is scale-invariant (JMP version 10, SAS Institute Inc., Carry, NC, USA). Logistic regression analysis was used to compare species specific survival among sites [44]. Differences in growth within and between species was compared among sites and position within the water-column using general linear mixed models, with a Tukey’s Honestly Significant Difference (HSD) post hoc analysis among sites [45]. Pearson’s correlation was used to examine the relationship between selected nutrient variables and river mile. P-values were reported and Type-I error (α) of 0.05 was used to judge significance of the statistical tests.

## Results

### Mussel survival and growth

**Fig 2:**
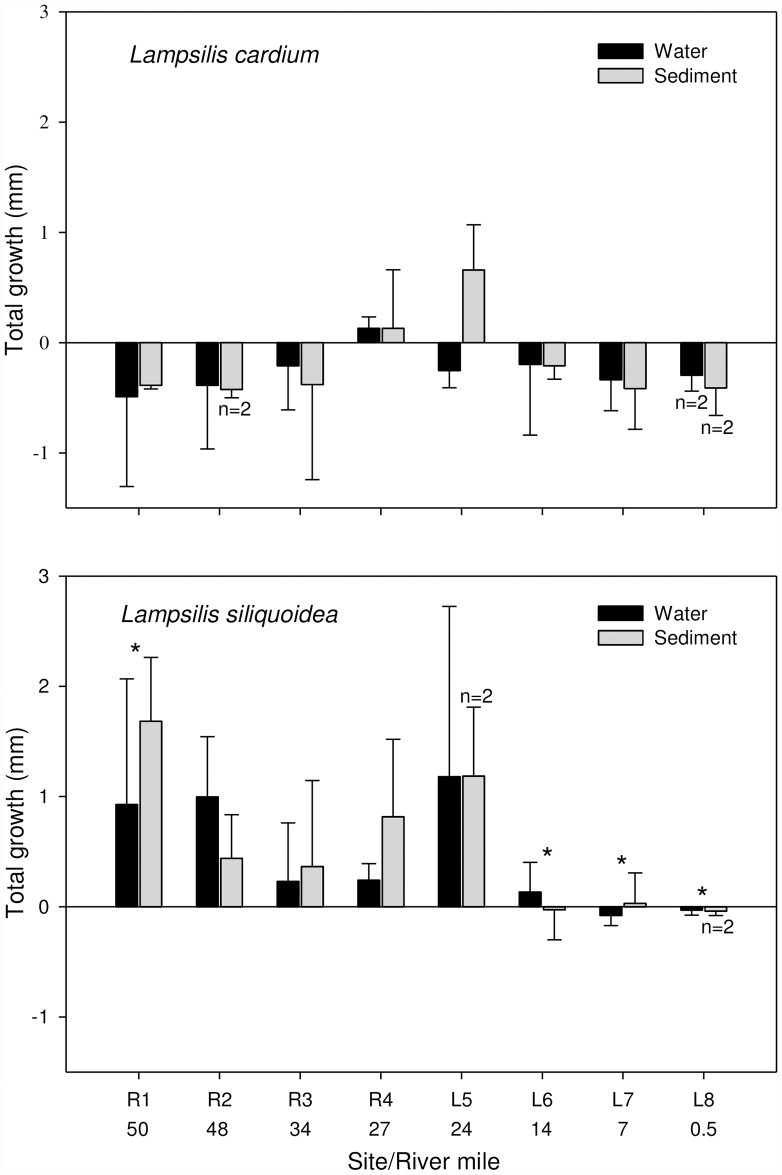
Mean growth (difference between the initial and final length) of juvenile *Lampsilis cardium* and *L*. *siliquoidea* deployed for 28 d at four riverine and four lacustrine sites in the St. Croix River. Unless noted n = 3. Error bars represent ± 1 SE. An * denotes significant differences, Tukey’s HSD, p <0.05.

We recovered all 48 *in situ* exposure cages and juvenile mussels that were deployed in the St. Croix River. Overall mussel survival was 95%, with four mussels dying in the lake (L) and one in the river (R). There were no differences in survival (p >0.56) or growth (p >0.51) of either mussel species deployed in the water-column compared to those held at the sediment-water interface (Fig 2). However, the growth of *L*. *siliquoidea* was significantly greater at R1 compared to L6 – 8 (p <0.05, Fig 2). *Lampsilis cardium* exhibited lower growth relative to *L*. *siliquoidea* (p <0.0001), but the growth of *L*. *cardium* was not significantly different across sites (p = 0.13).

### Lipid and fatty acid content in mussel foot tissue

**Fig 3:**
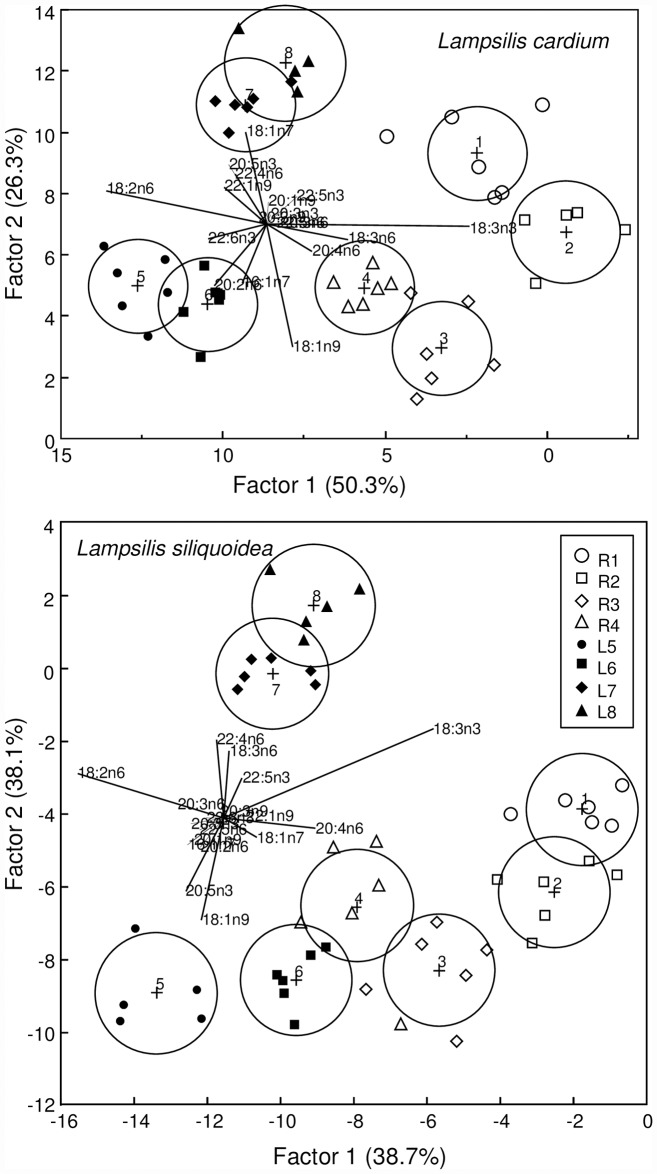
Canonical scores plot derived from a discriminant analysis of the concentrations of 18 fatty acids in the foot tissues of *Lampilis cardium* and *L*. *siliquoidea* grouped by site in the St. Croix River during a 28 d *in situ* exposure. Open and closed symbols are from juveniles deployed at riverine and lacustrine sites, respectively. The centroid for each 95% confidence region is denoted by a +.

**Fig 4:**
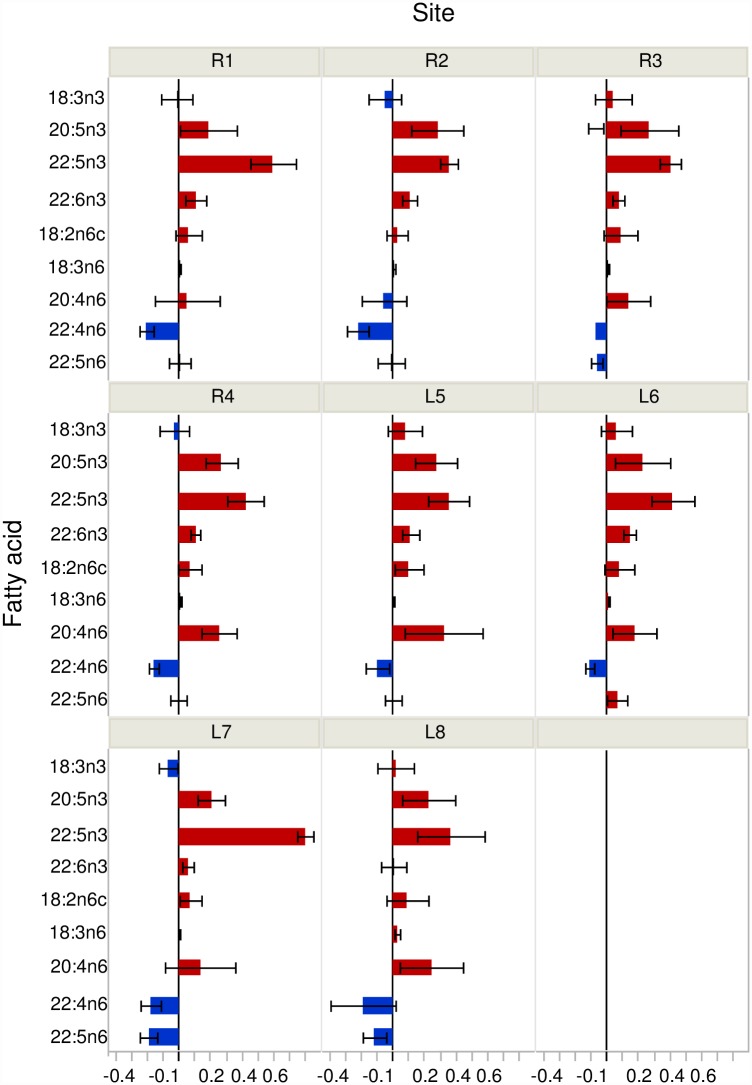
Differences in the concentration of selected fatty acids in the foot tissue of *Lampsilis cardium* and *L siliquoidea* located at a given site (i.e., four riverine or four lacustrine sites in the St. Croix River during August 2008). Positive values indicate a greater concentration in *L*. *siliquoidea* compared to *L cardium*. Error bars represent ± 1 SE.

**Fig 5:**
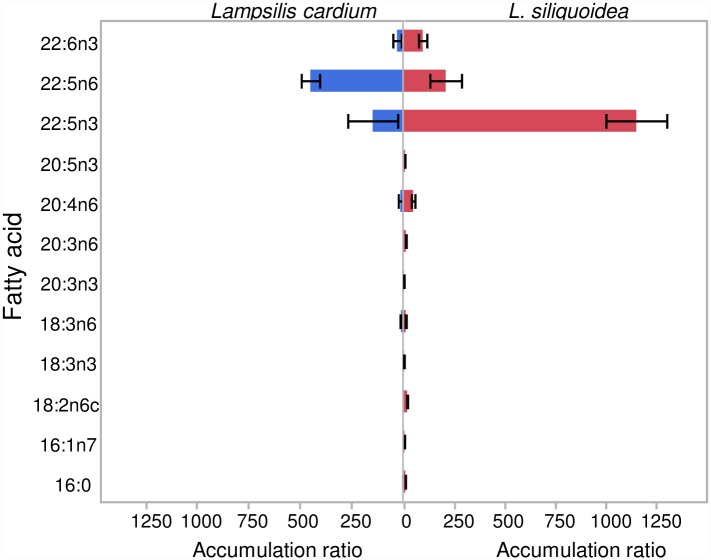
Fatty Acid Accumulation Ratios (FAAR) for *Lampsilis cardium* and *L*. *siliquoidea* held in a laboratory for 28-d and fed a commercial *Nannochloropsis* diet. FAAR represents the ratio of the 28-d net tissue accumulation of a specific fatty acid divided by the concentration of that fatty acid in the diet for a given mussel species.

The mean total lipid content (% dry weight) in *L*. *siliquoidea* was 9.1% (range 3.8 to 10.7%) and in *L*. *cardium* was 9.3% (range 6.8 to 11.4%). Total lipid content was not correlated with juvenile growth for either species (r = -0.07, p ≥0.60). However, discriminant analysis indicated that the FA profiles of the mussels were site specific and consistent among individuals allowing for site level discrimination (Fig 3), with a zero misclassification rate for both species. For *L*. *cardium*, factor 1 separated along the vectors of 18:2n6 to 18:3n3 and 18:3n6, whereas factor 1 separated along the vectors of 18:2n6 to 18:3n3 and 20:4n6 for *L*. *siliquoidea*. For *L*. *cardium*, factor 2 separated along the vectors 18:1n9 to 18:1n7 and 20:5n3; whereas for *L*. *siliquoidea* factor 2 separated along the vectors 18:1n9 and 20:5n3 to 22:4n6 and 18:3n6 (Fig 3). When comparing the two species at a given site, *L*. *siliquoidea* had greater concentrations of omega-3 FAs (i.e., 20:5n3, 22:5n3, 22:6n3) relative to *L*. *cardium*, whereas *L*. *cardium* had greater concentrations of 22:4n6 within their tissue (Fig 4). In our 28 d laboratory feeding experiment, the FA profiles of mussel foot tissue revealed that 22:5n3, 22:5n6, 22:6n3, and 20:4n6 had the greatest accumulation ratios (FAAR) relative to the commercial *Nannochloropsis* diet (Fig 5).

### Water column nutrients and sediment characteristics

**Fig 6:**
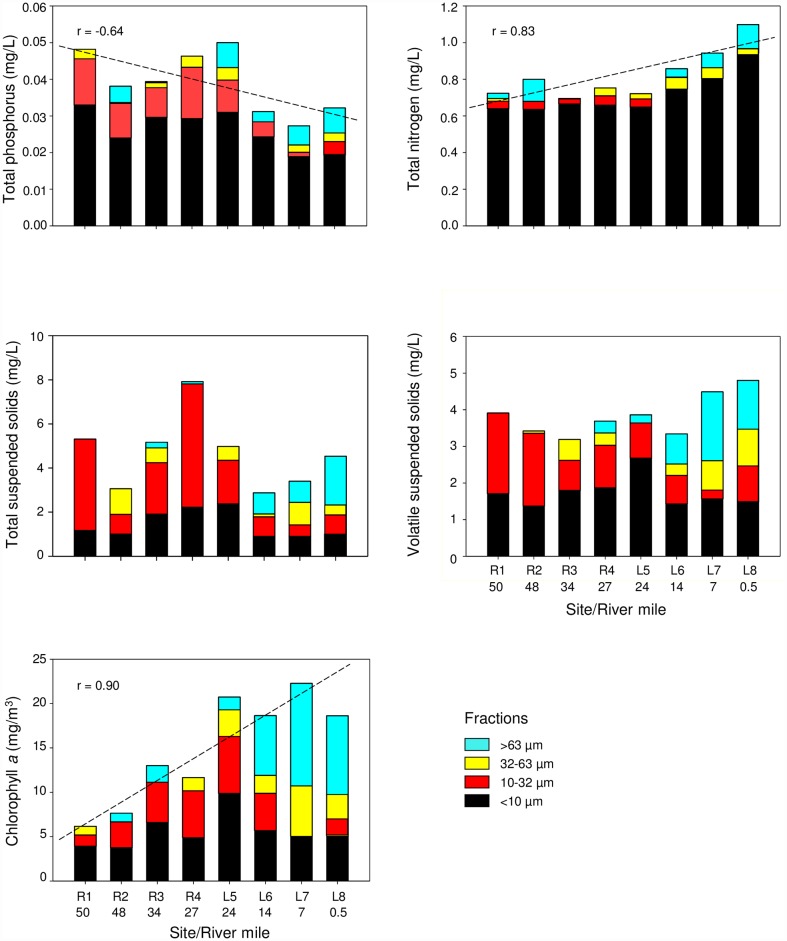
Total Phosphorus (TP, mg-P/L), Total Nitrogen (TN, mg-N/L), Total Suspended Solids (TSS, mg/L), Volatile Suspended Solids (VSS, mg/L), and chlorophyll *a* (mg/m^3^) in water samples by size fraction collected from four riverine and four lacustrine sites in the St. Croix River during August 2008. River mile indicated under site designation.

**Fig 7:**
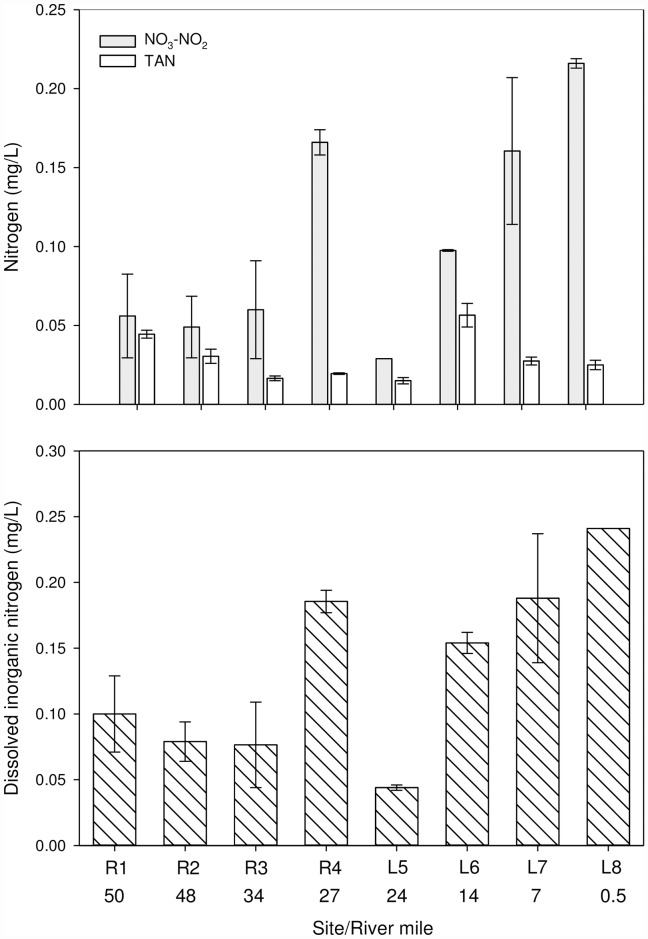
Nitrate-Nitrite (NO_3_-NO_2_, mg-N/L), Total Ammonia Nitrogen (TAN, mg-N/L), and Dissolved Inorganic Nitrogen (DIN, mg-N/L) in water samples collected (n = 2 unless noted) from four riverine and four lacustrine sites in the St. Croix River during August 2008. Error bars represent the range.

Total phosphorus (TP) concentrations in whole water from the river to the lake (r = -0.64, p <0.001), with the majority of the TP being associated with the <10 μm fraction (Fig 6). Soluble reactive phosphorus ranged from 0.008 to 0.012 mg/L (near the limit of quantification, 0.005mg/L) and also tended to decrease from the river to lake sites (r = -0.75, p <0.001). In contrast, total nitrogen (TN) concentrations in whole water increased downstream (r = 0.83, p <0.001, Fig 6). Similar to TP, the majority of the TN was associated with the <10 μm fraction (Fig 6). The nitrate-nitrite (NO_3_-NO_2_) concentrations were highest downstream of agricultural tributaries: the Kinnickinnic (RM6, 0.22 mg/L) and Apple (RM30, 0.17 mg/L) Rivers; whereas, the concentration of total ammonia nitrogen (TAN) was highest below the Willow River (RM18, 0.06 mg/L, Fig 7). Dissolved inorganic nitrogen (DIN) concentrations ranged from 0.04 to 0.24 mg/L, with the lowest concentration occurring at the upper end of Lake St. Croix, near Stillwater (RM24), then increasing downstream to Prescott (RM0.3, Fig 7).

Total suspended-solids (TSS) concentrations in whole water did not suggest a spatial trend (Fig 6). The greatest TSS concentration was at R4, below the Apple River tributary (RM30), whereas the lowest concentration was at R2. However, TSS concentrations generally increased from the river to the lake in the >63 μm fraction (r = 0.83, p <0.0001, Fig 6). In the riverine sites, the majority of TSS was associated with the <32 μm fraction (Fig 6). Volatile suspended-solids (VSS) concentrations in whole water ranged from 3.2 to 4.8 mg/L. The greatest VSS concentration was at L8 near Prescott (Fig 6). Similar to TSS concentrations, the VSS concentrations generally increased from the river to the lake in the >63 μm fraction (r = 0.87, p <0.0001, Fig 6).

The carbon (C) content in lake sediments was generally higher than river sites, except L6 (Table 2), whereas the nitrogen (N) content did not differ upstream to downstream. The carbon to nitrogen ratio in the sediments ranged from 10.4 to 21.2 in the river sites and from 10.0 to 68.6 in the lake sites. Concentrations of TAN and unionized ammonia nitrogen (NH_3_-N) in the sediment pore water varied by site (Table 2). In the riverine sites, TAN ranged from 0.14 to 1.04 mg/L, whereas the lacustrine sites ranged from 0.01 to 3.07 mg/L. Concentrations of NH_3_-N ranged from 2.7 to 9.6 μg/L and 0.08 to 23.7 μg/L in the river and lake sites, respectively.

**Table 2:** Sediment and pore water characteristics (mean ± 1 SE, n = 3) at four riverine and four lacustrine sites on the St. Croix River during a 28 d *in situ* juvenile mussel exposure.

	Sediment			Pore water^a^		
Site	Carbon (%)	Nitrogen (%)	C:N ratio	TAN (mg/L)	NH_3_-N (μg/L)	NO_3_-NO_2_
R1	0.39 ± 0.18	0.02 ± 0.00	21.20 ± 8.50	1.04 ± 0.32	9.61 ± 3.09	0.03 ± 0.00
R2	0.22 ± 0.01	0.02 ± 0.00	11.33 ± 0.35	0.14 ± 0.02	3.40 ± 0.37	0.04 ± 0.01
R3	0.14 ± 0.02	0.02 ± 0.00	10.42 ± 0.24	0.20 ± 0.05	2.70 ± 0.54	0.03 ± 0.00
R4	0.15 ± 0.03	0.01 ± 0.00	12.69 ± 0.34	0.38 ± 0.00*	4.06 ± 1.05*	0.03 ± 0.00*
L5	1.38 ± 0.52	0.05 ± 0.02	29.24 ± 5.34	3.07 ± 0.13	15.26 ± 0.32	0.03 ± 0.00
L6	0.18 ± 0.03	0.02 ± 0.00	10.03 ± 0.76	0.01 ± 0.00	0.08 ± 0.02	3.34 ± 0.04
L7	1.59 ± 0.23	0.03 ± 0.00	68.57 ± 7.58	3.07 ± 0.28	23.71 ± 2.92	0.05 ± 0.01
L8	1.16 ± 0.25	0.02 ± 0.00	63.04 ± 2.09	0.72 ± 0.21	12.03 ± 2.47	0.07 ± 0.01

^a^The detection limit for total ammonia nitrogen (TAN) was 0.004 mg/L. Unionized ammonia (NH_3_-N) was calculated from site and replicate-specific TAN, pH and temperature measurements. The detection limit for nitrate-nitrite (NO_3_-NO_2_) was 0.029 mg/L. Means with * have percent difference reported as the error, n = 2.

### Biotic characteristics—Chlorophyll and phytoplankton

**Fig 8:**
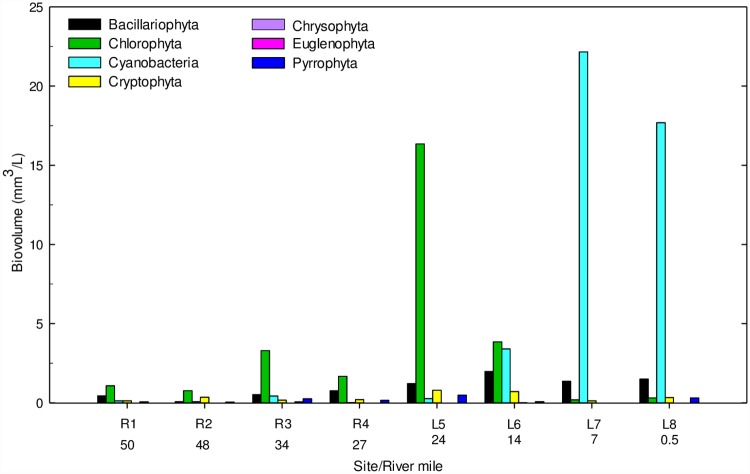
Biovolume (mm^3^/L) of phytoplankton in whole water samples collected from four riverine and four lacustrine sites in the St. Croix River during August 2008.

Chlorophyll *a* concentrations in whole water increased from the river to the lake and ranged from 3.8 to 21.9 mg/m^3^ (r = 0.90, p <0.0001, Fig 6). The riverine sites, including L5, had greater concentrations of chlorophyll *a* in the <32 μm fractions, whereas L6-8 had greater chlorophyll *a* concentrations in the >63 μm fraction (Fig 6). Seventy-seven algal taxa were identified: 12 diatom taxa, 39 greens, 15 cyanobacteria, seven cryptophytes, one chrysophyte, one euglenophyte, and two dinoflagellate taxa. Diatoms were observed at all sites but generally less abundant in the river (range 0.07 to 0.75 mm^3^/L) compared to the lake sites (range 1.21 to 1.98 mm^3^/L, Fig 8). *Aulacosiera granulata*, *Stephanodiscus niagarae*, *Synedra delicatissima*, *Melosira varians* were abundant in riverine sites, whereas, *Aulacosiera granulata* and *Fragilaria crotonensis* were abundant in lacustrine sites. Green algae were abundant in the river (range 0.77 to 3.29 mm^3^/L) and reached their highest level at L5 (16.35 mm^3^/L, Fig 8). *Pediastrum duplex*, *Oocystis borgei*, *Chlamydomonas* spp., and *Crucigenia crucifera* were abundant in the riverine sites, whereas, *Pediastrum duplex*, *Nephrocytium agardhianum*, and *Eudorina spp*. were abundant at lacustrine sites. Cyanobacteria were also identified at all sites; and ranged from 0.03 to 0.43 mm^3^/L and 0.27 to 22.16 mm^3^/L in the river and lake sites, respectively (Fig 8). *Microcystis wesenbergii*, *Woronichinia naegeliana*, and *Synechecoccus* spp. were abundant in riverine site, whereas, *Aphanizomenon flos-aquae*, *Microcystis aeruginosa*, *Dolichospermum spiroides*, and D. *circinale* were abundant at lacustrine sites.

### Growth of *Lampsilis siliquoidea*—Relation to nutrients and available food resources

**Fig 9:**
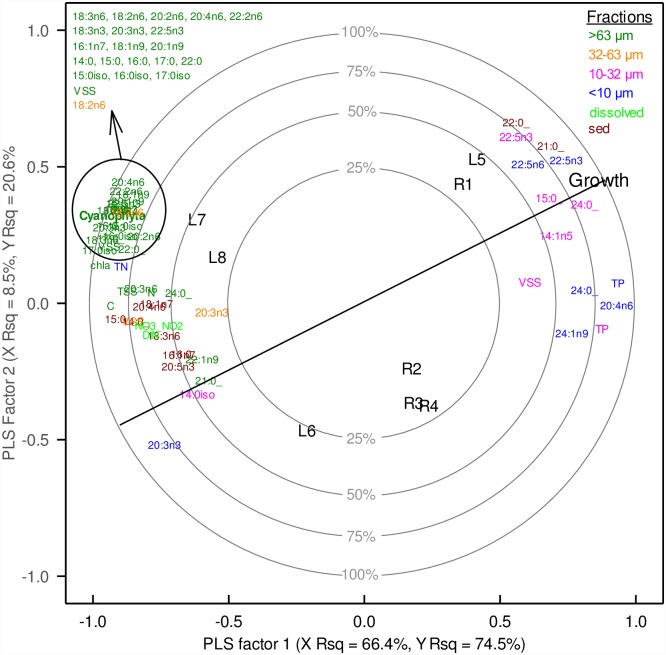
Correlation loading plot for the Partial Least Squares (PLS) Regression of *Lampsilis siliquoidea* growth. The plot is derived from a reduced set of predictor variables having a Variable Important to Projection (VIP) value ≥1.25 in the full model. The sampling sites (i.e., observations, labeled R1 – R4 for riverine and L5 – L8 for lacustrine) are located relative to their X score for each PLS factor. The location of the sites orthogonal to the vector (black line) labeled “Growth” indicate the relative magnitude of the growth response (e.g., L5 and R1 having highest growth with sites L6, L7, L8 exhibiting low or no growth). Variables located nearer the label “Growth” along the diagonal line passing through the origin, are highly correlated with growth relative to those variables located opposite of growth, which are negatively related to growth. The majority of the variation in “Growth” was explained by PLS factor 1 (YR^2^ = 74.5%), with an additional 20.6% of variation explained by PLS factor 2. The combined variation explained by model factors 1 and 2 is 94.5%.

**Fig 10:**
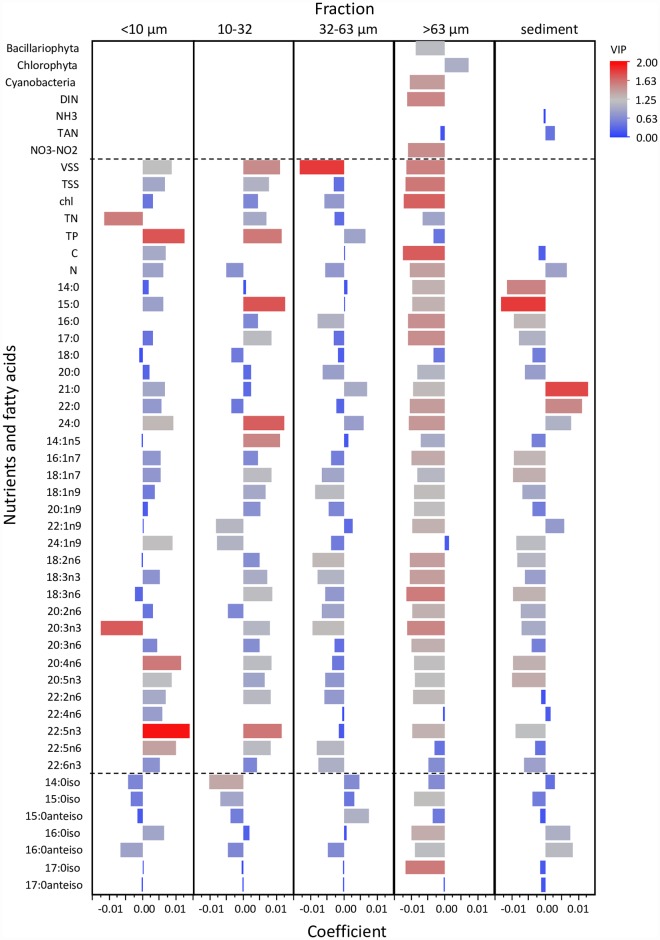
Partial Least Squares (PLS) model coefficients for water column and sediment variables used to predict *Lampsilis siliquoidea* growth. The direction (+ or -) and length of the horizontal bars relative to the origin indicate the relationship and strength of these predictors to *L*. *siliquoidea* growth based on centered (mean = 0) and scaled (standard deviation = 1) data for the full PLS model. The bar coloration specifies Variable Importance to Projection (VIP), such that red bars indicate higher VIP scores and increased contribution to the overall model for both the predictor and the response variables.

The partial least squares regression (PLSR) analysis and the resulting correlation loading plot (Fig 9) explained the differences in the growth response of *L*. *siliquoidea*. The first two latent factors explained >95% of the variability in growth (ΣR^2^Y) and ~75% of the predictor variation (ΣR^2^X, Fig 9). Sites with the greatest average mussel growth (L5 and R1) are located near the “Growth” response variable (see Fig 9), whereas sites with low growth (L6 and L7, L8) are located opposite growth and orthogonal to the line passing through the origin. Growth of *L*. *siliquoidea* was greater in sites with greater concentrations of TP in the <10 and 10 – 32 μm fractions, VSS in the 10 – 32 μm fraction and several FAs associated with the seston; the PUFAs, 22:5n3 (<10 μm and 10 – 32 μm fractions), 20:4n6 and 22:5n6 (<10 μm fraction); two saturated FAs, 15:0 (10 – 32 μm fraction) and 24:0 (<10 and 10 – 32 μm fractions); and two FAs associated with the sediments, 21:0 and 22:0 (Figs 9 and 10). In contrast, growth of *L*. *siliquiodea* was less in sites with greater concentrations of NO_3_-NO_2_, DIN, TN in the <10 μm fraction and nearly all FAs in the largest size fraction (i.e., >63 μm) of seston, including the bacterial FAs, and several of the FAs associated with the sediments (Figs 9 and 10). Growth of *L*. *siliquoidea* was less in sites with greater concentrations of particulate C, N, TSS, VSS and chlorophyll *a* within the >63 μm fraction (Figs 9 and 10). In addition, *L*. *siliquoidea* growth was less in sites where the abundance of cyanobacteria exceeded 9% of the total phytoplankton biovolume.

## Discussion

**Fig 11:**
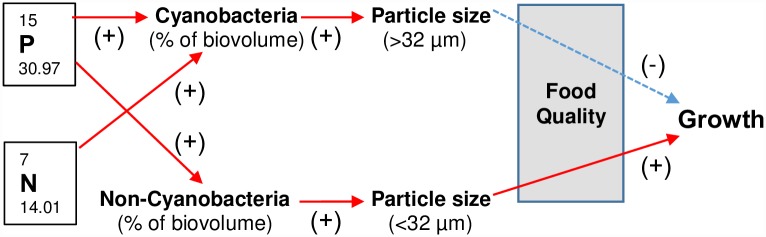
Conceptual framework relating predictor variables to observed patterns in patterns in growth. Red arrows imply positive relationship, blue arrows indicate a negative relationship.

Our data strongly suggest that mussel growth in the St. Croix River system is affected by the size and quality of the available food resources. We offer a conceptual framework that summarizes the specific factors that likely influence mussel growth in this system (Fig 11). Overall, the nutrient availability (N and P) influenced the standing stock and composition of the phytoplankton community along this riverine-lacustrine gradient. An increase in TN mid-river (site L5) along with an increased abundance of cyanobacteria corresponded with change in the quality and particle-size of food resources. The ability of mussels to acquire sufficient food resources is dependent on their ability to remove, sort, and retain size specific, high quality food items from the seston. Dominance of colonial forming cyanobacteria in the plankton community, redistributes the autotrophic biomass from the <32 μm size class to the larger size classes, this may present a food resource challenge. Mussels are typically thought to retain particles <32 μm in diameter [3], therefore, the quality of the particles within this size range are important for growth and survival.

### Nutrients and flow affect phytoplankton composition

Our study reach functioned as a riverine — transition — lacustrine reservoir system [46, 47]. The riverine sites (R1 – 4) were shallow and turbulent, with high levels of VSS in the <32 μm fraction and fairly consistent N and P concentrations, resulting in a phytoplankton community that was dominated primarily by green algae (range 58 to 70% total biovolume) and secondarily by diatoms (range 5 to 28% total biovolume). The riverine sites also exhibited a longitudinal increase of chlorophyll *a* which reached a maximum concentration at the uppermost lake site (L5). This site served as a transition zone, where chlorophyll *a* concentration peaked and then generally plateaued throughout the lacustrine sites (L6 – 8). In contrast, TN steadily increased within the lacustrine sites, resulting in an increased abundance of cyanobacteria.

The transition zone was characterized by high TP concentrations, high levels of VSS in the <10 μm fraction, and a biovolume (mm^3^/L) concentration more than 3 fold higher than the riverine sites. Green algae were the most abundant taxa within the transition zone (site L5, 85% total biovolume) as well, which likely resulted from increased light availability due to the settling of inorganic suspended solids and moderate levels of turbulence compared to the more riverine sites. Transition zones, such as site L5, represent biological hot spots for phytoplankton production and/or biogeochemical processing [46] and provide an optimal set of conditions to support high algal biomass. Others have found high densities and growth rates of mussels at lake outlet streams [48] and they attributed this in part to the flux of nutrients from the river to the lake generating additional food supply. At this site, the phytoplankton were dominated by a diverse assemblage of green algae (including 19 species), many of which have a diameter or major axis of <20 μm size. By far, the most abundant green algae at site L5 was *Pediastrum duplex* (~64% of total biolvolume), which has been shown to compete poorly with cyanobacteria under low turbulence (e.g., *Microcystis*; [49]). Within the nutrient replete environment of this transition zone, cyanobacteria would be expected to dominate the phytoplankton community [46, 50] if not for mediating effects of turbulence. Huisman et al. [49] demonstrated through both theoretical modeling and empirical field experiments that in turbulent systems, sinking phytoplankton species, both diatoms (e.g., *Asterionella*) and green alga (e.g., *Pediastrum*), were able to out compete the buoyant cyanobacterium *Microcystis*; however, under low or non-turbulent conditions *Microcystis* often prevailed. In addition, within this transition zone, both nitrate and DIN concentrations were markedly lower, likely because of phytoplankton uptake; whereas, TP concentrations were at the maximum and exhibited a moderate decline throughout Lake St. Croix. Dzialowski et al. [47] observed a similar decline in TP in Kansas reservoirs, where the loss of inorganic P bound to non-algal sediment particles settled out in a transitional zone, and thereby reduced the P available for the phytoplankton. The cause for the P decline at sites L6 – 8 is unknown, but may indicate a settling loss of phytoplankton or a loss of P bound to inorganic solids.

### Importance of particle size and taxonomic composition

Freshwater mussels filter a limited size range of particles and food-handling time may play an important role in the ingestion process. Most of the seston in the riverine sites were associated with the <32 μm fraction; whereas lacustrine sites (except L5) had a larger proportion of seston biomass in the >63 μm fraction. Nichols and Garling, [3] determined that mussels required food in the <32 μm fraction, given this size range, then on average 11% to 47% of the phytoplankton for riverine and lacustrine sites, respectively, would be in a form too large for ingestion based on our estimates of the seston suspended organic fraction. The lacustrine sites (L7 – L8) had the highest proportion of seston in the larger size fractions (>32 μm) and these sites were dominated by cyanobacteria. Laboratory studies have shown that freshwater mussels are capable of selective feeding. Bontes et al. [51] determined that *Anodonta anatine* were able to discriminate between food sources and preferentially selected the green alga, *Scenedesmus obliquus*, over filamentous and coccoid cyanobacteria (*Planktothrix agardhii* and *Microcystis aeruginosa*, respectively), as evidenced by higher pseudofaeces production upon ingestion of the cyanobacteria. Beck and Neves [52] observed evidence that *Villosa iris* selectively feed on the basis of particle size, such that smaller particles of *Nannochloropsis oculata* and *Selenastrum capricornutum* (~3 to 9 μm size range) are preferred over larger colonies of *Scenedesmus quadricauda* (22 to 45 μm size range). These studies suggest that particle size and composition of available food resources may limit filter-feeding bivalves.

### Quality of cyanobacteria as a food resource

Cyanobacteria are regarded as a low-quality food in terms of quality and size structure for higher trophic levels. For example, Heathcote et al. [53] reported that zooplankton biomass was two orders of magnitude less than would be predicted based on phytoplankton biomass in hypereutrophic lakes. In these highly productive systems, the phytoplankton community became increasingly dominated by cyanobacteria (>89% of phytoplankton biomass), which lead to an increase in the particle size of the resulting phytoplankton community. The redistribution of the phytoplankton biomass into larger size classes resulted in a higher proportion of the standing stock unavailable to zooplankton grazers, and thus limited their productivity. In a 28-d feeding study, the somatic growth rate of juvenile *Corbicula fluminea* was significantly lower when fed cyanobacteria (*Aphanizomenon flos-aquae*, *Anabaena variabilis*, *Synechococcus elongatus*) diets than juveniles fed algae (*Scenedesmus obliquus*, *Cryptomonas* sp.), which they attributed in part to the lack of long-chained PUFAs and select sterols within the cyanobacteria diets [18]. In our study, both species of juvenile mussels exhibited reduced growth within the lacustrine sites (L7 – L8) and these sites were dominated by cyanobacteria (93% and 88% of the total biovolume, respectively). Furthermore, certain species of cyanobacteria are capable of producing toxins [54]. The most abundant cyanobacteria in the Lake St. Croix were *Aphanizomenon flos-aquae*, *Dolichospermum* spp (*D*. *circinale*, *D*. *spiroides*), and *Microcystis aeruginosa*, and all of these species are known to produce cyanotoxins [54]. Freshwater mussels may accumulate high concentrations of these toxins within their tissue; although not found to be acutely toxic, sublethal effects on growth were not assessed [55].

### Growth of *Lampsilis siliquoidea* in relation to *in situ* food resources

Few studies have documented the *in situ* FA composition of native freshwater mussels [56–58] and none have specifically characterized the FA composition within the available food resources. In our study, *L*. *siliquoidea* growth was positively related to several PUFAs in the smaller seston size fractions. For example, the omega-3 PUFA 22:5n3 (docosapentaeonic acid, DPA, in the <10 μm and 10 – 32 μm fractions) was positively related to juvenile mussel growth and this FA is in the pathway of EPA (20:5n3) and DHA (22:6n3), both of which have been shown to be essential for survival and growth of marine bivalves [59]. Additionally, mussel growth was positively related to greater concentrations of two omega-6 PUFAs, 20:4n6 and 22:5n6 (<10 μm fraction), and both freshwater and marine mussels have been shown to selectively retain 20:4n6 [11, 57, 60]. In freshwater mussels, the retention of 20:4n6 may have important physiological functions, such as regulation of Na uptake [60]. Recently, Strandberg et al. [61] demonstrated that *Daphnia magna* was capable of retroconverting dietary 22:5n6 to 20:4n6. If mussels are also capable of retroconverting dietary 22:5n6 to 20:4n6, then this may partially explain the discrepancies between the FA composition in the phytoplankton (i.e., food source) and that of the mussel tissue. In our laboratory feeding experiment, we observed that 22:5n3, 22:5n6, 22:6n3, and 20:4n6 had the highest accumulation ratios (FAAR) relative to the mussel diet. In the case of 22:5n3, this suggests that mussels either have a high retention preference for this FA or that the mussels are capable of elongating 20:5n3 into 22:5n3; however, this has yet to be confirmed. The correspondence of high FAAR ratios in laboratory-held *L*. *siliquoidea* and those deployed *in situ* indicates that 22:5n3 and 22:5n6 are physiologically important FAs.

There were also two saturated FAs within the seston (15:0, 10 – 32 μm fraction and 24:0, <10 and 10 – 32 μm fractions) and two within the sediment (21:0 and 22:0) that were positively related to *L*. *siliquoidea* growth. While we are not advocating that saturated FAs are essential for growth, they do occur in freshwater mussel tissues [57, 58]. Of the four saturated FAs, 15:0 is considered a bacterial marker and 22:0 and 24:0 are terrestrial markers [11]. The positive association of these saturated FAs with *L*. *siliquoidea* growth may indicate a utilization of bacterial and terrestrial derived carbon within the mussel diet.

Previous bivalve studies have made comparisons of the FA composition in resident organisms of the same or similar species at different locations as a means of assessing nutritional or physiological condition [56, 57]. One of the fundamental challenges we face is the lack of knowledge of the basic requirements for a given species. For example, if specific FAs (e.g., 20:5n3, 22:6n3, 20:4n6) are important for growth and survival of unionid mussels, then at what concentration are they limiting? There are no definitive values reported in the literature for comparison. Here, we experimentally relocated two species of juvenile mussels, previously held under similar conditions, into both riverine and lacustrine habitats and compared their FA composition after a 28-d deployment and assessed survival and growth. We used juveniles because of their fast growth rate, limited food reserves, and the likelihood that they were not reproductively active. This design likely enhanced our ability to assess site-specific effects of food quality on mussel growth. Our study was one of the first to document the rapid incorporation of a unique, site specific FA profile (i.e., 28-d deployment) within freshwater mussel tissue; however, the tissue profile was not predictive of growth.

### Differences in tissue fatty composition between *Lampsilis* species

Overall, *L*. *siliquoidea* had a higher n3:n6 ratio relative to *L*. *cardium* (range, 0.67 to 1.03 and 0.63 to 0.90 for *L*. *siliquoidea* and *L*. *cardium*, respectively) and this difference was primarily due to greater concentrations of 20:5n3 (EPA) and 22:5n3 (DPA) in *L*. *siliquoidea*; while, *L*. *cardium* had greater concentrations of 22:4n6 across all sites. The differences in the FA composition between two closely related mussel species is notable and although not related to growth, may indicate subtle differences in feeding strategies or sensitivity to disturbance. Our exposure cages were initially positioned at the sediment-water interface; however, due to the hydrology of the river and the sifting nature of the sediments, not all sediment-water interface cages remained in contact with the sediments throughout the 28 d duration. If *L*. *cardium* is more dependent on the sediment for proper orientation and optimal feeding compared to *L*. *siliquoidea*, this may result in differential filtration rates and corresponding fatty acid composition between the two species. Kryger and Riisgård [62] determined that the filtration rate of four bivalve species was higher when they were in their natural position with about 2/3 of the body buried in sediment compared to those lying on one valve outside of the sediment. The filtration rate of undisturbed mussels (i.e., buried in sediment) was found to be at least four times higher than previously reported. Interestingly, in a laboratory study, the filtration rates of adult *Lampsilis radiata siliquoidea* buried in sediment compared to those lying on one valve were not significantly different [5], suggesting that positioning for optimal feeding may not be as critical for *L*. *r*. *siliquoidea* compared to other species. The definitive cause for the differences in the fatty acid composition between these two species requires further investigation.

### Conclusions

This study has shown that local environmental conditions can affect the quality of food available for juvenile mussels in the St. Croix River. *Lampsilis siliquoidea* growth was positively related to VSS (10 – 32 μm fraction), TP (<10 and 10 – 32 μm fractions) and select FA in the seston (22:5n3, 22:5n6, 20:4n6, and 24:0 in the <10 μm fraction and 22:5n3, 15:0 and 24:0 in the 10 – 32 μm fraction) and the sediment (C22:0). In contrast, growth of *L*. *siliquiodea* was negatively related to nearly all FAs in the largest size fraction (i.e., >63 μm) of seston, including the bacterial FAs, and several of the FAs associated with the sediments. Although we were able to demonstrate that juvenile mussels acquire a site specific FA signature within 28 d, the FA content of their foot tissue did not predict growth. We suggest that to adequately understand the food resources available for mussel growth, we need to better understand the partitioning of food resources into size fractions that the mussels can ingest and metabolize. It is the combination of high quality food, adequate size fraction, and abundance that likely effects mussel growth and survival. If increases in cyanobacteria populations do occur (as predicted by changing environmental conditions), then our results suggest that juvenile mussels may experience longer periods of food-limitation, ultimately leading to reduced growth and increased risk of mortality.
